# Supplementary material for: Energy comparison of sequential and integrated CO2 capture and electrochemical conversion
Source: Nat Commun. 2022 Sep 14;13:5398. doi: 10.1038/s41467-022-33145-8 (PMC9474516; doi:10.1038/s41467-022-33145-8)
Supplement: Supplementary file 1 — Supplementary Information [file 41467_2022_33145_MOESM1_ESM.pdf]

Supplementary Information

Energy comparison of sequential and integrated CO_2 capture and electrochemical conversion

Mengran Li[1], Erdem Irtem[1], Hugo-Pieter Iglesias van Montfort[1], Maryam Abdinejad[1], Thomas Burdyny*[1]

Materials for Energy Conversion and Storage (MECS), Department of Chemical Engineering, the Delft University of Technology, van der Maasweg 9, 2629 HZ Delft, The Netherlands.

Corresponding author: t.e.burdyny@tudelft.nl

Supplementary note 1

Estimation of energy consumption for the integrated electrolyser

A summary of variables definition and values for the equations below are given in Supplementary Table 1.

Anode potential

We assumed the anode reaction for the gas-fed electrolyser is to evolve oxygen from water in a basic environment (1 M KOH aqueous solution, pH = 14). The anode's overpotential is the same as the gas-fed CO_2 electrolysis process. The charge-transfer reactions at the anode can be described by the Tafel equation when the anode overpotential is larger than 200 mV:

$$j = j_o \times \left(\frac{c}{c^{ref}}\right)^{\gamma} \times exp\left(\frac{\alpha_a z_O F}{RT} \times \eta\right) \tag{1}$$

The overall anode potential can be calculated by:

$$E_{anode} = U + \eta \tag{2}$$

Ohmic loss

We assumed the electrode is far more electronically conductive than the membrane and electrolytes. The ohmic losses should be a sum of resistances at the ion-exchange membrane, catholyte and anolyte. The ohmic area specific resistance of the membrane or electrolytes can be determined by:

$$ASR_k = \frac{1}{\sigma_k} \times L_k \tag{3}$$

The ohmic loss can be estimated by:

$$\eta_{ohm} = \sum_k ASR_k \times j \tag{4}$$

Supplementary Figure 1 shows a sum of our estimated anode potential and ohmic loss based on our assumptions. (see Supplementary Table 1)

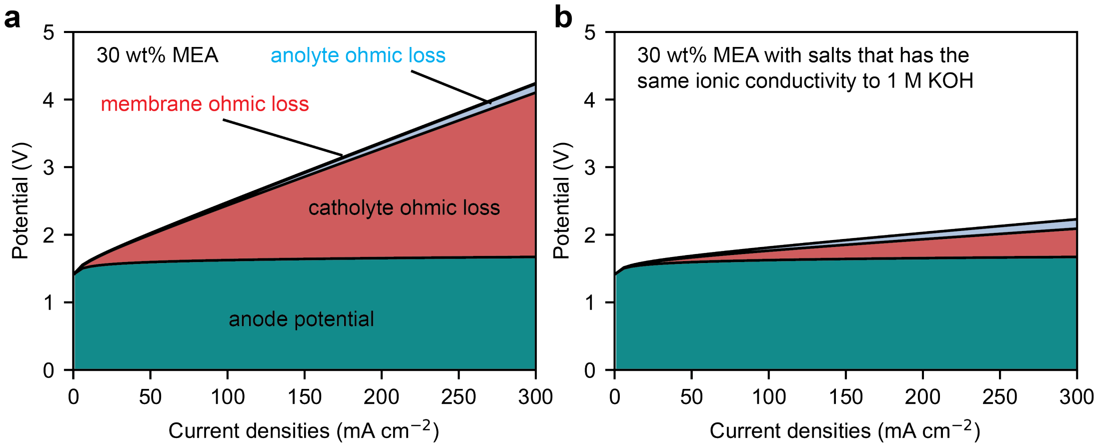

Supplementary Figure 1 Relation between current densities and potentials (without cathode potential) for the integrated CO_2 electrolyser with (a) 30 wt% monoethanolamine solution as the catholyte and (b) 30 wt% monoethanolamine solution with inorganic salts as the catholyte that has the same ionic conductivity (21.5 S m^{-1}) for the 1 M KOH.

The overall cell voltage can be estimated by:

$$E_{cell} = E_{anode} - E_{cathode} + \eta_{ohm} \quad (5)$$

The cathode potentials were extracted from literature data summarized in Supplementary Table 2. The Nernstian overpotential, defined as the potential related to the pH differences at cathode and anode, was not considered in this work.

The energy consumed to convert 1 mol CO_2 to CO electrochemically can be calculated by:

$$Q = \frac{E_{cell} \times j \times z \times F}{FE_{CO} \times j} \quad (6)$$

<u>Estimation of energy consumption for gas-fed CO_2 electrolyser</u>

We estimated the energy consumption by using the Eq.1-7, where the cell voltages were reported in recent literature, summarized in Supplementary Table 3.

Supplementary Table 1 Summary of variables definition and values for supplementary note 1

Variables	Description	Value and/or unit
j	Current density (mA cm^{-2})	-
j_o	Exchange current density (mA $^{-2}$)	0.01 mA cm^{-2} Ref[1]
$\left(\frac{c}{c_{ref}}\right)^{\gamma}$	Concentration-related terms, where c is concentration of OH^- to be oxidized, and c_{ref} is the reference concentration, γ is the reaction order.	In 1 M KOH aqueous anolyte, this ratio is unity. [2-5]
α_a	Anode charge transfer coefficient	0.6 Ref[1]
z_O	The number of charge to produce one oxygen molecule	4
F	Faraday constant	96485 s A mol^{-1}
R	Gas constant	8.314 J mol^{-1} K^{-1}
T	Temperature	273 K
η	Overpotential of anode	V
A	Pre-exponential factor	1.23×10^{-4} mA cm^{-2} [2-5]
E_a	Apparent activation energy	25 kJ mol^{-1} [2-5]
E_{anode}	Anode potential	V vs SHE
U	Equilibrium potential	1.23 V vs. SHE
ASR_k	Area specific ohmic resistance of k component (catholyte, anolyte or Nafion membrane)	Ω cm^2
σ_k	Electrical conductivity of the k component at 25 °C	30 wt% MEA (5 M) has an ion conductivity of 3.7 S m^{-1} ; Ref[6]<br>1 M KOH has an ion conductivity of 21.53 S·m^{-1} Ref[7]<br>Nafion membrane conductivity is assumed to be 8 S m^{-1} Ref[8]
L_k	The length of the ion transport of component k	Ion transport length in anolyte was assumed to be 1 mm;<br>Ion transport length in catholyte was assumed to be 3 mm;<br>Thickness of the Nafion 115 is 115 μm.

η_{ohm}	Ohmic loss	V
E_{cell}	Cell voltage	V
$E_{cathode}$	Cathode potential	V vs. RHE
Q	Energy convert n to convert 1 mol CO_2 to CO	kJ per mol CO_2
FE_{CO}	Faradaic efficiency of CO	%
z	The number of electrons to produce single CO molecule from CO_2	2

Supplementary Table 2 Summary of recent reports on electrochemical CO_2 reduction directly from concentrated amine solutions

Cathode	Cathode Potential (V) vs. RHE	FE_{CO} (%)	Current densities (mA cm^{-2})	Solvents	Ref
Ag/carbon-black on 300 nm Ag film on ePTFE	-0.8	72	50	30wt% MEA mixed with 2M KCl with no dissolved CO_2	9
Ag/carbon-black on 300 nm Ag film on ePTFE	-1.2	20	100	30 % wt. MEA mixed no dissolved CO_2	9
Ag foil	-0.8	12.4	10*	30 wt% MEA	10
Ag foil	-1.1	6.1	10*	30 wt% MEA	10
Ag foil	-1.3	2.3	10*	30 wt% MEA	10
Ag foil	-0.8	33.4	10*	30wt% MEA with 0.1% wt/wt CTAB	10
Ag foil	-1.1	15.9	10*	MEA with 0.1% wt/wt CTAB	10
Ag foil	-1.3	9.2	10*	30wt% MEA with 0.1% wt/wt CTAB	10
Cu	-0.78	45	18.4	0.1mM ethylenediamine carbamate in 0.1M $NaClO_4$	11
Smooth Au foil	-1.9 vs. Ag|AgCl	45	15	2-amino-2-methyl-1-propanol (AMP) and propylene carbonate (PC) solution	12

Au/MgAl-LDHs	-0.4	68	0.8	1.0 M alcohol amine solution (n(ethanolamine): n(diethanolamine) = 2:3)	13
Cu/MgAl-LDHs	-0.25	73	0.4	1.0 M alcohol amine solution(n(ethanolamine): n(diethanolamine) = 2:3)	13
Ag	-1.1	71	15	[MEAHCl][MDEA], where MEAHCl is ethanolamine hydrochloride, and MDEA is methyl diethanolamine	14

* These values are our estimations because the original paper did not report the exact values.

Supplementary Table 3 Summary of examples of normal CO_2 electrolysis to produce CO.

Cathode	Cathode Potential (V) vs RHE	Cell Voltage (V)	FE_{CO} (%)	Current densities (mA cm^{-2})	Ref
Ag nanoparticles on GDL Sigracet 39BC	n/a	3.1	87	556.7	15
Ag nanoparticles on GDL Sigracet 35BC	n/a	3	95	200	16
Ag nanoparticles GDE	n/a	3	97.5	120	17
Ag nanoparticles on GDL Sigracet 35BC	n/a	3	98	200	17
Ag nanoparticles on GDL Sigracet 35BC	n/a	3.1	95	400	17
Ag nanoparticles on GDL Sigracet 35BC	n/a	3.3	96	600	17
Au/C AvCard GDS3250		3	85	500	18
100 nm Ag on Sigracet 39BC	-0.45	3.4	81.5	300	19
Ag nanoparticles/MWCNT on Sigracet 35BC	-0.72	3	95	340	20

Supplementary note 2

Sequential route

The flow diagram of the sequential route is shown in Supplementary Figure 2.

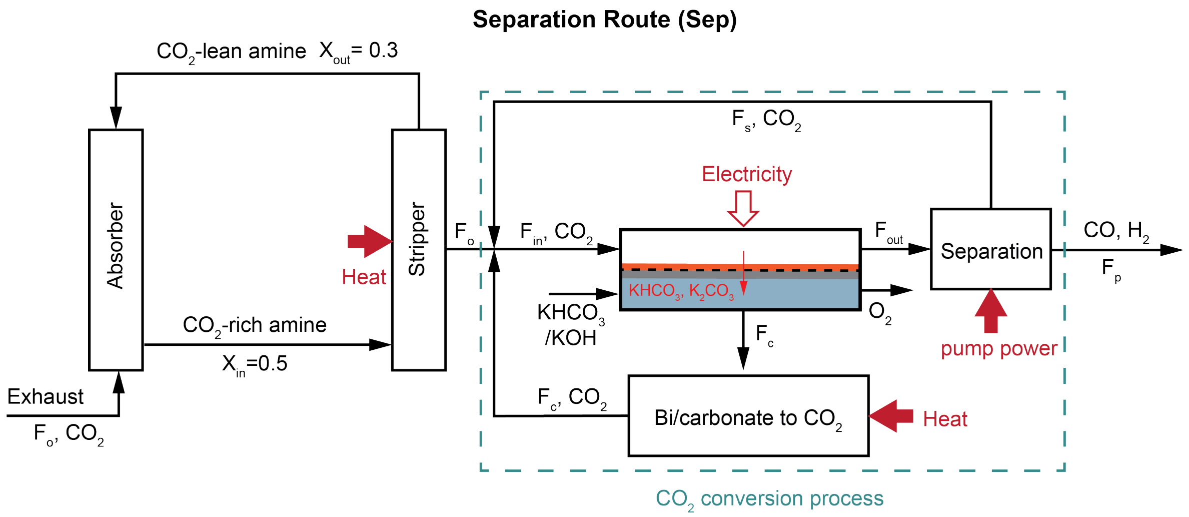

Supplementary Figure 2 A flow diagram of separation of CO_2 capture and electrolysis process to produce CO.

<u>Key assumptions</u>

The following are the key assumptions for the carbon balance.

CO_2 electrolysis process

- The electrolysis cell voltage is 3 V with a CO Faradaic efficiency of 90%. This assumption can be validated by Supplementary Table 3.
- We assumed the gas-fed electrolyser has a 50% CO_2 utilisation efficiency, meaning for 1 mol CO_2 conversion to CO, 1 mol CO_2 would be lost in the formation of (bi)carbonate. The CO_2 loss in (bi)carbonate is due to the high local pH at the catalyst surface, which leads to the homogenous reactions between OH^- and CO_2. (see reactions (7) – (9)) This phenomenon has been reported in recent literatures.[21] Although this critical issue can be addressed by introducing protons close to the catalyst surface (e.g., using acidic catholyte,[22,23] cation-exchange membrane,[24] and bipolar membrane[25]), in this work we chose the widely reported CO_2 electrolyser containing alkaline catholytes (e.g., KOH) or based on membrane-electrode assemblies in the sequential route.

$$CO_2 + H_2O + 2e^- \rightarrow CO + 2OH^- \quad (7)$$

$$CO_2 + 2OH^- \rightarrow CO_3^{2-} + H_2O \quad (8)$$

$$CO_2 + OH^- \rightarrow HCO_3^- \quad (9)$$

- The formed (bi)carbonate does not evolve CO_2 in the electrolyser. There are studies reporting the detection of CO_2 from the anode chamber likely due to the acidification at the anode, but such process will cause increased anode overpotential. For simplicity, we assumed all the formed (bi)carbonates will be regenerated in the regeneration unit downstream.

- The formed (bi)carbonates will be regenerated to become CO_2 for electrolysis. The energy for the regeneration is assumed to be 254 kJ mol^{-1}, including 231 kJ mol^{-1} heat duty and 23 kJ mol^{-1} electricity. [21,26]
- The product of CO_2 conversion is the mixture of CO and H_2, which can be the feedstock for the manufacturing processes such as syngas in the Fischer-Tropsch process.
- The electrolyser has a single-pass overall CO_2 conversion rate of 50 %, meaning that 25 mol% of CO product and 25 mol% of (bi)carbonate formation.

Amine scrubbing unit

- This work limits the scope to the traditional monoethanolamine-based scrubbing process due to the widely reported data and demonstration of pilot plants in the past decades.[27-32]
- The amine scrubbing unit for the downstream separation requires similar energy to recover CO_2 from amine solutions.
- The CO_2 loading in the CO_2-rich amine stream from the absorption column is 0.5 mol CO_2 per mol amine, and the CO_2-lean stream contains 0.3 mol per mol amine.[33,34]
- The heat duty to separate CO_2 from amine solutions in the stripper is between 88 – 203 kJ mol^{-1} if there is heat integration in the process.[30,32,35,36] The baseline is assumed to be 179 kJ mol_{CO2}^{-1} to reflect a higher TRL proof of concept.
- Compression of the CO_2 after CO_2 recovery requires 14 - 19 kJ mol^{-1}.

Overall process

- A pressure swing adsorption process is assumed to separate CO_2 from the electrolyser cathode gas effluent because the pressure-swing adsorption only requires 14.5 – 36.4 kJ per mol CO_2.[37-39]
- This work is only focused on evaluating the dominant energies involved in this sequential route, which includes the heat to regenerate the amine, the heat for electrolyser gas product separation, and electrical energy for the CO_2 conversion. Other energies required for pump, compressor, cooling and hot water utilities are not considered in our model.
- The thermal energy price is assumed to be \$2.69 per million British Thermal Units[40], and the electricity price is assumed to be \$0.04 per KWh.[41]

<u>Carbon balance</u>

$$F_{in} - F_{out} - F_c = 0 \tag{10}$$

$$F_{in} = F_o + F_s + F_c \tag{11}$$

$$F_{out} = F_s + F_p \qquad (12)$$

We assumed the single pass conversion x of CO_2 in the electrolyser is 0.5, so F_{in} and F_s indicate the CO_2 inlet and outlet flow rates, respectively. Therefore, the CO_2 balance is:

$$F_{in} - F_s = F_{in}x \qquad (13)$$

We assumed that the overall process is at steady state and all the CO_2 captured are converted to CO.

$$F_o = F_p \qquad (14)$$

The CO_2 utilisation efficiency is assumed to 50%, so the carbonation rate and CO production rate is same.

$$F_c = F_p \qquad (15)$$

Combining Eq. 2-1, 2-4, 2-5, 2-6 yields the influent flow rate:

$$F_{in} = \frac{2F_o}{x} \qquad (16)$$

Combining Eq. 2-4 and 2-7 yields the CO_2 recovery rate from separation process.

$$F_s = 2F_o(\frac{1}{x} - 1) \qquad (17)$$

Energy analysis

Energy for amine regeneration and CO_2 recovery:

$$Q_{ar} = Q_{sar} \times \frac{F_o}{F_o} \qquad (18)$$

$$Q_{psa} = Q_{sspa} \times \frac{F_s}{F_o} \qquad (19)$$

The equivalent work for the amine regeneration can be calculated from:

$$W_{ar} = \epsilon_{turbine} \times \frac{T_{stm} - T_{sink}}{T_{stm}} \times Q_{ar} \qquad (20)$$

Because of $F_c = F_o$ in our assumption, the energy to recover one molar CO_2 from the (bi)carbonate is:

$$Q_c = Q_{sc} \times \frac{F_c}{F_o} \qquad (21)$$

Energy required for CO_2 electrolysis:

$$Q_e = \frac{E \times j}{j \times FE/(z \times F)} \times \frac{F_o}{F_o} \qquad (22)$$

Energy required for product separation:

$$Q_{psa} = Q_{spsa} \times \frac{F_s}{F_o} \tag{23}$$

The sum of the key energies for the sequential route is:

$$Q_{sequential} = Q_{ar} + Q_{compression} + Q_c + Q_e + Q_{psa} \tag{24}$$

If there is no carbonate formation, then the conversion rate x is the conversion rate of CO_2 to CO product. There are following changes in calculating F_s and Q_{sep}.

$$F_s = F_o(\frac{1}{x} - 1) \tag{25}$$

$$Q_{psa} = Q_{spsa} \times \frac{F_s}{F_o} \tag{26}$$

$$Q_{sequential} = Q_{ar} + Q_{compression} + Q_e + Q_{psa} \tag{27}$$

Integrated route

The flow diagram of the integrated route is shown in Supplementary Figure 3.

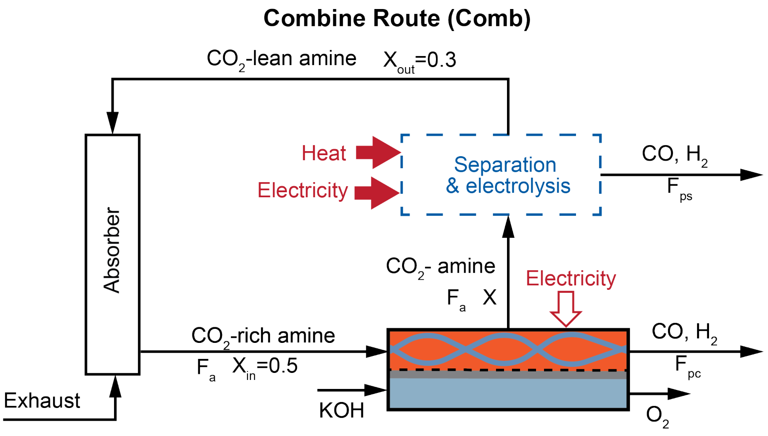

Supplementary Figure 3 A schematic flow diagram for the integrated route between CO_2 capture and electrolysis.

Key assumptions

We made a few assumptions as follows to simplify our calculations.

Electrolyser

- The electrolyser directly reduces CO_2-rich amine stream from the absorber to CO gas product.

- There is no carbonate formation in the electrolyser because there are no gaseous CO_2 in the electrolyser to react with OH^-.
- Considering the mass transfer limitations, we assume the electrolyser can only achieve a CO_2 loading of X in the effluent of the electrolyser.
- The amine solution is stable during electrolysis. Lee et al.[9] recently demonstrated the ability of amine recycling after CO_2 electrolysis.

Separation

- To maintain the same CO_2 loading in the amine solutions, a separation and electrolysis process was included. This process includes amine regenerations, CO_2 gas-fed flow-cell electrolyzer, carbonate regeneration, and downstream separation process. This process simplifies our model to estimate the maximum energy penalty due to a higher X than X_{out}.

Amine absorption unit

- We assumed both separation and integrated routes have the same absorption unit in the capture process.

<u>Carbon balance</u>

The following equation calculates the flow rate of CO_2 in amine solution that is converted in the CO_2 electrolyser.

$$F_{pc} = F_a \times (X - X_{in}) \tag{28}$$

The flow rate of the rest CO_2 to the separation & electrolysis process is

$$F_{ps} = F_a \times (X - X_{out}) \tag{29}$$

<u>Energy analysis</u>

The energy required to convert one mole CO_2 in the amine solutions in the integrated electrolysis:

$$Q_{e\_integrated} = \frac{F_a}{F_a} \times \frac{X_{in} - X}{X_{in} - X_{out}} \times \frac{E_{integrated} \times j}{j \times FE_{integrated}/(z \times F)} \tag{30}$$

The energy required to capture and convert the CO_2 in the normal electrolyser in the separation & electrolysis process:

$$Q_{e\_integrated\_s} = \frac{F_a}{F_a} \times \frac{X - X_{out}}{X_{in} - X_{out}} \times \frac{E \times j}{j \times FE/(z \times F)} \tag{31}$$

The energy required for amine regeneration in the overall process is:

$$Q_{ar\_integrated} = \frac{F_a}{F_a} \times Q_{sar} + Q_{ar} \times \frac{F_a}{F_a} \times \frac{X - X_{out}}{X_{in} - X_{out}} \tag{32}$$

The energy required for (bi)carbonate regeneration process is:

$$Q_{c\_integrated} = \frac{F_a}{F_a} \times \frac{X - X_{out}}{X_{in} - X_{out}} \times Q_c \tag{33}$$

The sum of the key energies for the integrated route is:

$$Q_{integrated} = Q_{ecomb} + Q_{ecombs} + Q_{arcomb} + Q_{ccomb} \tag{34}$$

Supplementary Table 4 lists all the variables used in the carbon balance and energy analysis. The energy cost can be calculated by:

$$Cost_{thermal} = Q_{thermal} \times \frac{p_{thermal}}{10^6}/1.055 \tag{35}$$

$$Cost_{electricity} = Q_{electricity} \times \frac{p_{electricity}}{3600 \times 44} \times 10^6 \tag{36}$$

Supplementary Table 4 Summary of the variables and their definitions

Symbol	Definition	Values or unit
F_{in}	Influent of the electrolyser	mol CO_2 h^{-1}
F_{out}	Effluent of the electrolyser	mol C h^{-1}, should include flow rates of CO_2 and CO in the stream
R	The total reaction rate in the electrolyser	mol CO_2 h^{-1}
F_c	The rate of (bi)carbonate formation	mol CO_2 h^{-1}
F_s	The flow rate of CO_2 recovered from the downstream separation process	mol CO_2 h^{-1}
F_p	The production rate of the CO product	mol CO_2 h^{-1}

F_o	The flow rate of CO_2 from the stripper unit	mol CO_2 h^{-1}
x	Single-pass conversion rate of the electrolyser	We assumed to be 0.5, meaning that 0.25 for CO_2 to CO, and 0.25 for CO_2 to (bi)carbonate
F_{ar}	The total flow rate of CO_2 recovered from the amine regeneration	mol CO_2 h^{-1}
Q_{ar}	Total energy required for amine regeneration including heat integration	We assumed the range is at 88 – 203 kJ per mol CO_2. The baseline value is 179 kJ per mol CO_2 to reflect higher TRL proof of concept
Q_{sar}	Specific energy required to recover 1 mol CO_2 from amine regeneration	kJ per mol CO_2
Q_{psa}	The energy required to recover CO_2 from the product stream using pressure swing adsorption	kJ per mol CO_2
$Q_{compression}$	The energy required to compress CO_2 after recovery	We assumed the range is at 14 – 19 kJ per mol CO_2. The baseline value is chosen as 16.5 kJ per mol CO_2.
Q_{spsa}	Specific energy required to recover one mole CO_2 from the product stream using pressure swing adsorption	We assumed the range is at 14.5 ~ 36.4 kJ per mol CO_2. The baseline value is chosen as 25.46 kJ per mol CO_2.
Q_c	Total energy required to recover CO_2 from (bi)carbonate	We assumed the baseline value is at 254 kJ per mol CO_2.
Q_{sc}	Energy to recover 1 mol CO_2 from (bi)carbonate	kJ per mol CO_2
Q_e	Energy required to convert 1 mol CO_2 in MEA-based electrolyser	kJ per mol CO_2
z	The number of charges transferred to convert 1 CO_2 molecule to CO molecule.	z = 2 for CO production
F	Faraday constant	96485 C mol^{-1}
j	Current densities of the CO_2 electrolyser	mA cm^{-2}
E	Cell voltage	V
$Q_{sequential}$	Total energy required to convert one mole CO_2 in the sequential route	kJ per mol CO_2
X_{in}	The CO_2 loading in the effluent of the absorption unit	We assumed 0.5 mol CO_2 per mol amine.

X_{out}	The CO_2 loading in the CO_2-lean amines.	We assumed the CO_2-lean amine contains 0.3 mol CO_2 per mol amine.
F_a	Molar flow rate of amine in the effluent of the absorption unit	mol amine h^{-1}
F_{pc}	Flow rate of the CO_2 that is converted in the integrated electrolyser.	mol CO_2 h^{-1}
X	CO_2 loading in the effluent of the integrated electrolysis	We assumed 0.3 mol CO_2 per mol amine in our study as the baseline value.
F_{ps}	The flow rate of CO_2 that is converted in the separation & electrolysis process	mol amine h^{-1}
$Q_{e\_integrated}$	The energy required to convert CO_2 in the amine solutions to CO in the integrated electrolyser.	kJ per mol CO_2
$Q_{e\_integrated\_s}$	The energy required to convert CO_2 to CO in CO_2 electrolyser in the separation & electrolysis process.	kJ per mol CO_2
$Q_{ar\_integrated}$	The energy required to recover CO_2 from the amine solutions in the integrated route.	kJ per mol CO_2
$Q_{c\_integrated}$	The energy required to recover CO_2 from (bi)carbonates in the integrated route.	kJ per mol CO_2
$Q_{integrated}$	The sum of key energies in the integrated route.	kJ per mol CO_2
$Cost_{thermal}$	The energy cost associated with thermal energy consumption	\$ ton_{CO2}^{-1}
$Q_{thermal}$	Thermal energy requirement for the process.	kJ mol_{CO2}^{-1}, the thermal energy is associated with amine regeneration in the CO_2 capture step and calcination step in the (bi)carbonate regeneration.
$p_{thermal}$	The price of the thermal energy.	We assumed the price of thermal energy from natural gas is $2.69 per million British thermal units. [40] The current price is unusually higher due to the Russia-Ukraine conflict.
$Cost_{electricity}$	The energy cost associated with the electricity.	\$ ton_{CO2}^{-1}
$Q_{electricity}$	The energy requirement associated with the electric work.	kJ mol_{CO2}^{-1} se electric energy associated with compressor, PSA separation, electrolysis, and (bi)carbonate regeneration.

$p_{electricity}$	The price of the electricity.	The electricity price is assumed to be \$0.04 per kWh. [41]

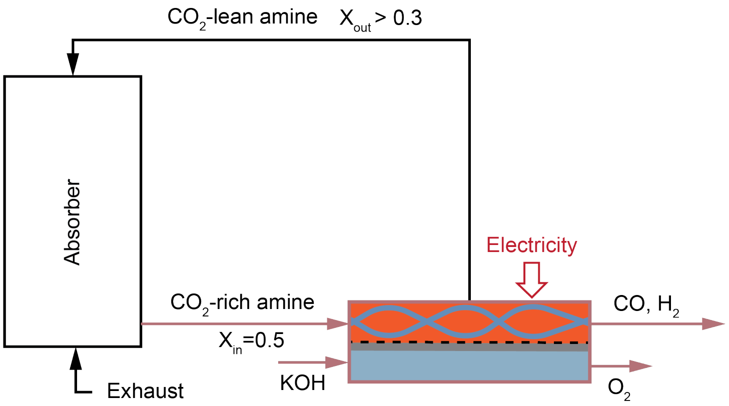

Supplementary Figure 4 A schematic illustration of the integrated route that requires a sizeable absorber to account for the incomplete conversion in the integrated electrolysis system.

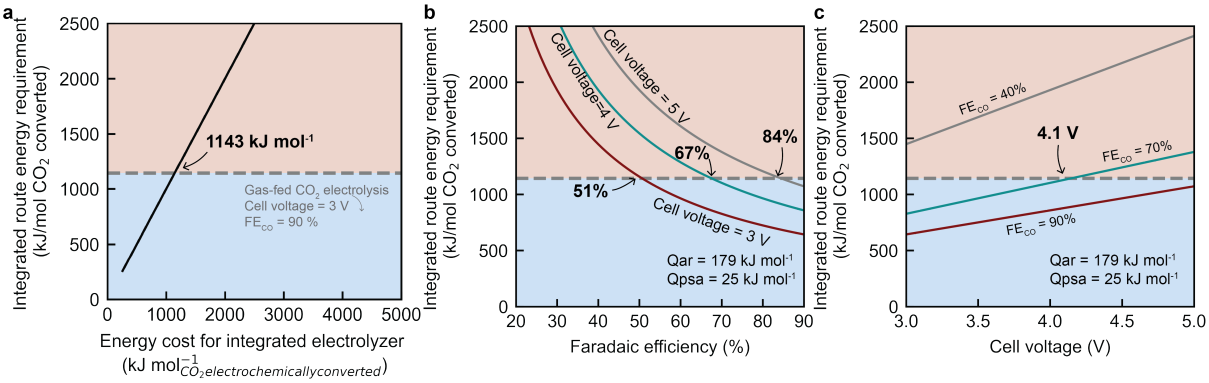

Supplementary Figure 5 Impacts on overall integrated energy cost from integrated electrolysers. **a,** Effect of energy cost **b,** CO FE at a cell voltage at 3, 4, and 5 V and **c,** cell voltages with CO Faradaic efficiency (FE) of 40%, 70%, and 90% on the overall energy cost of the integrated route. The grey dashed line represents the energy cost of the sequential route based on state-of-the-art gas-fed CO_2 electrolysers. The blue region means that the integrated route is more energy-efficient than the sequential route.

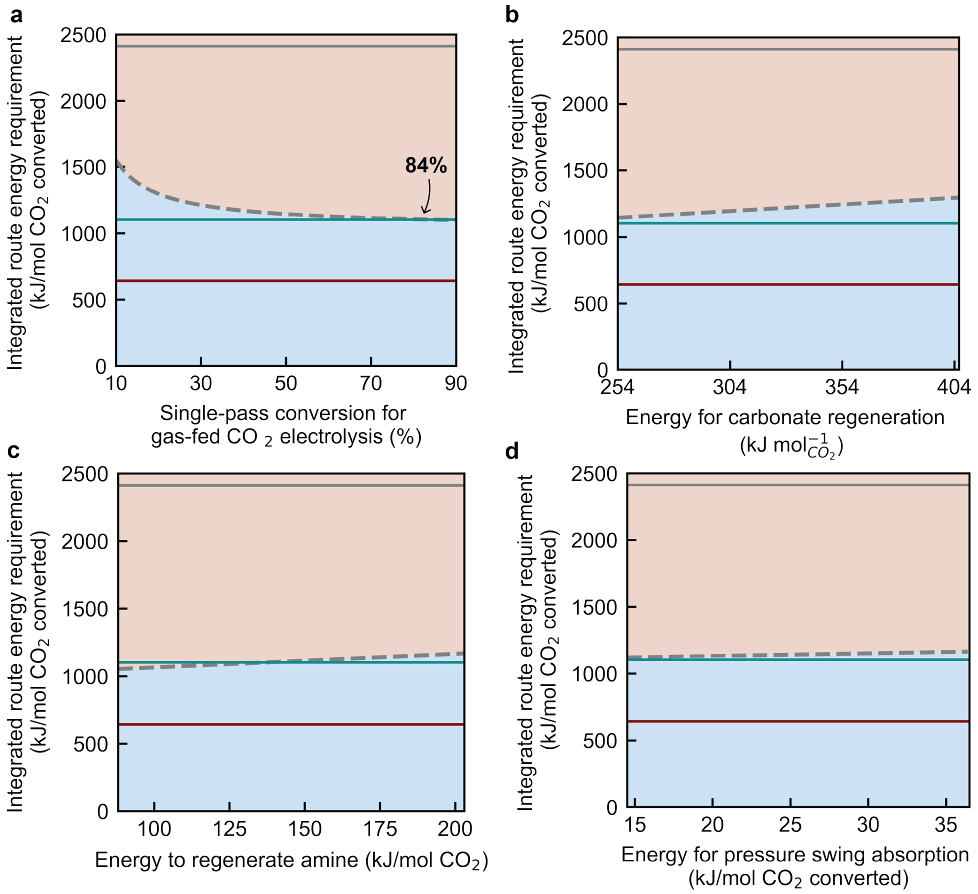

Supplementary Figure 6 Effects of operating conditions and solvent properties on the overall energy costs of the sequential and integrated route. The overall energy cost of the sequential route and integrated route as a function of **a,** single-pass conversion for gas-fed CO_2 electrolysis (note that the single-pass conversion is the ratio of total CO_2 consumed vs. the CO_2 feed), **b,** CO_2 loading in the effluent of the integrated electrolyser from capture media in the sequential route, **c,** energy required to regenerate amine-based capture medium with heat integration included, and **d**, the energy required to separate product from the effluent stream of gas-fed CO_2 electrolysers. The figures show baseline (green), pessimistic (grey), and optimistic (red) scenarios of the integrated route. The blue region is where the integrated route has energy advantages, while the orange region is vice versa.

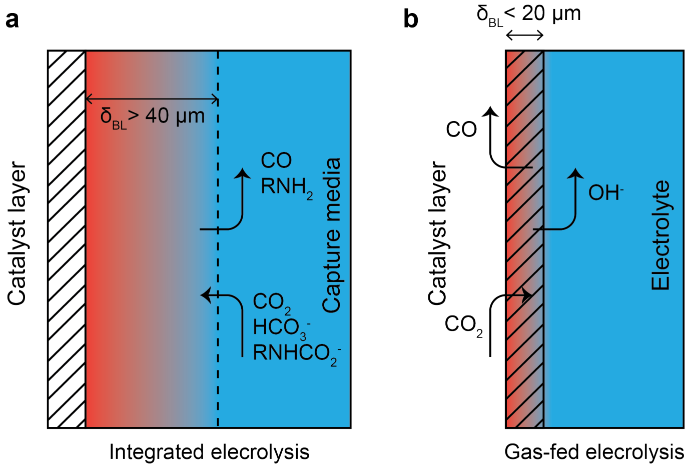

Supplementary Figure 7 Comparison of the thickness of hydrodynamic boundary layers (δ_{BL}) at the cathode for (a) integrated electrolysis if the cell configuration is similar to gas-fed H-cell and (b) gas-fed electrolysis.

References

1 McCallum, C. *et al.* Reducing the crossover of carbonate and liquid products during carbon dioxide electroreduction. *Cell Reports Physical Science* **2**, 100522, doi:https://doi.org/10.1016/j.xcrp.2021.100522 (2021).

2 Weng, L.-C., Bell, A. T. & Weber, A. Z. Towards membrane-electrode assembly systems for CO2 reduction: a modeling study. *Energy & Environmental Science* **12**, 1950-1968, doi:10.1039/C9EE00909D (2019).

3 Kuo, D.-Y. *et al.* Influence of Surface Adsorption on the Oxygen Evolution Reaction on IrO2(110). *Journal of the American Chemical Society* **139**, 3473-3479, doi:10.1021/jacs.6b11932 (2017).

4 Nurlaela, E., Shinagawa, T., Qureshi, M., Dhawale, D. S. & Takanabe, K. Temperature Dependence of Electrocatalytic and Photocatalytic Oxygen Evolution Reaction Rates Using NiFe Oxide. *ACS Catalysis* **6**, 1713-1722, doi:10.1021/acscatal.5b02804 (2016).

5 Giordano, L. *et al.* pH dependence of OER activity of oxides: Current and future perspectives. *Catalysis Today* **262**, 2-10, doi:https://doi.org/10.1016/j.cattod.2015.10.006 (2016).

6 Han, S.-J. & Wee, J.-H. Estimation of the Amount of CO2 Absorbed by Measuring the Variation of Electrical Conductivity in Highly Concentrated Monoethanolamine Solvent Systems. *Journal of Chemical & Engineering Data* **61**, 712-720, doi:10.1021/acs.jced.5b00178 (2016).

7 Gilliam, R. J., Graydon, J. W., Kirk, D. W. & Thorpe, S. J. A review of specific conductivities of potassium hydroxide solutions for various concentrations and temperatures. *International Journal of Hydrogen Energy* **32**, 359-364, doi:https://doi.org/10.1016/j.ijhydene.2006.10.062 (2007).

8 Liu, L., Chen, W. & Li, Y. An overview of the proton conductivity of nafion membranes through a statistical analysis. *Journal of Membrane Science* **504**, 1-9, doi:https://doi.org/10.1016/j.memsci.2015.12.065 (2016).

9 Lee, G. *et al.* Electrochemical upgrade of CO2 from amine capture solution. *Nature Energy* **6**, 46-53, doi:10.1038/s41560-020-00735-z (2021).

10 Chen, L. *et al.* Electrochemical Reduction of Carbon Dioxide in a Monoethanolamine Capture Medium. *ChemSusChem* **10**, 4109-4118, doi:https://doi.org/10.1002/cssc.201701075 (2017).

11 Abdinejad, M., Mirza, Z., Zhang, X.-a. & Kraatz, H.-B. Enhanced Electrocatalytic Activity of Primary Amines for CO2 Reduction Using Copper Electrodes in Aqueous Solution. *ACS Sustainable Chemistry & Engineering* **8**, 1715-1720, doi:10.1021/acssuschemeng.9b06837 (2020).

12 Pérez-Gallent, E., Vankani, C., Sánchez-Martínez, C., Anastasopol, A. & Goetheer, E. Integrating CO2 Capture with Electrochemical Conversion Using Amine-Based Capture Solvents as Electrolytes. *Industrial & Engineering Chemistry Research* **60**, 4269-4278, doi:10.1021/acs.iecr.0c05848 (2021).

13 Li, L., Yang, J., Li, L., Huang, Y. & Zhao, J. Electrolytic reduction of CO2 in KHCO3 and alkanolamine solutions with layered double hydroxides intercalated with gold or copper. *Electrochimica Acta* **402**, 139523, doi:https://doi.org/10.1016/j.electacta.2021.139523 (2022).

14 Ahmad, N. *et al.* Electrochemical CO2 reduction to CO facilitated by MDEA-based deep eutectic solvent in aqueous solution. *Renewable Energy* **177**, 23-33, doi:https://doi.org/10.1016/j.renene.2021.05.106 (2021).

15 Endrődi, B. *et al.* Operando cathode activation with alkali metal cations for high current density operation of water-fed zero-gap carbon dioxide electrolysers. *Nature Energy* **6**, 439-448, doi:10.1038/s41560-021-00813-w (2021).

16 Kutz, R. B. *et al.* Sustainion Imidazolium-Functionalized Polymers for Carbon Dioxide Electrolysis. *Energy Technology* **5**, 929-936, doi:https://doi.org/10.1002/ente.201600636 (2017).

17 Kaczur, J. J., Yang, H., Liu, Z., Sajjad, S. D. & Masel, R. I. Carbon Dioxide and Water Electrolysis Using New Alkaline Stable Anion Membranes. *Frontiers in Chemistry* **6**, doi:10.3389/fchem.2018.00263 (2018).

18 Yin, Z. *et al.* An alkaline polymer electrolyte CO2 electrolyzer operated with pure water. *Energy & Environmental Science* **12**, 2455-2462, doi:10.1039/C9EE01204D (2019).
19 Gabardo, C. M. *et al.* Combined high alkalinity and pressurization enable efficient CO2 electroreduction to CO. *Energy & Environmental Science* **11**, 2531-2539, doi:10.1039/C8EE01684D (2018).
20 Ma, S. *et al.* Carbon nanotube containing Ag catalyst layers for efficient and selective reduction of carbon dioxide. *Journal of Materials Chemistry A* **4**, 8573-8578, doi:10.1039/C6TA00427J (2016).
21 Rabinowitz, J. A. & Kanan, M. W. The future of low-temperature carbon dioxide electrolysis depends on solving one basic problem. *Nature Communications* **11**, 5231, doi:10.1038/s41467-020-19135-8 (2020).
22 Huang, J. E. *et al.* CO₂ electrolysis to multicarbon products in strong acid. *Science* **372**, 1074-1078, doi:doi:10.1126/science.abg6582 (2021).
23 Monteiro, M. C. O., Philips, M. F., Schouten, K. J. P. & Koper, M. T. M. Efficiency and selectivity of CO2 reduction to CO on gold gas diffusion electrodes in acidic media. *Nature Communications* **12**, 4943, doi:10.1038/s41467-021-24936-6 (2021).
24 O'Brien, C. P. *et al.* Single Pass CO2 Conversion Exceeding 85% in the Electrosynthesis of Multicarbon Products via Local CO2 Regeneration. *ACS Energy Letters* **6**, 2952-2959, doi:10.1021/acsenergylett.1c01122 (2021).
25 Pătru, A., Binninger, T., Pribyl, B. & Schmidt, T. J. Design Principles of Bipolar Electrochemical Co-Electrolysis Cells for Efficient Reduction of Carbon Dioxide from Gas Phase at Low Temperature. *Journal of The Electrochemical Society* **166**, F34-F43, doi:10.1149/2.1221816jes (2019).
26 Keith, D. W., Holmes, G., St. Angelo, D. & Heidel, K. A Process for Capturing CO2 from the Atmosphere. *Joule* **2**, 1573-1594, doi:https://doi.org/10.1016/j.joule.2018.05.006 (2018).
27 Zhang, Y. & Chen, C.-C. Modeling CO2 Absorption and Desorption by Aqueous Monoethanolamine Solution with Aspen Rate-based Model. *Energy Procedia* **37**, 1584-1596, doi:https://doi.org/10.1016/j.egypro.2013.06.034 (2013).
28 Li, K. *et al.* Systematic study of aqueous monoethanolamine-based CO2 capture process: model development and process improvement. *Energy Science & Engineering* **4**, 23-39, doi:https://doi.org/10.1002/ese3.101 (2016).
29 House, K. Z. *et al.* Economic and energetic analysis of capturing CO₂ from ambient air. *Proceedings of the National Academy of Sciences* **108**, 20428-20433, doi:10.1073/pnas.1012253108 (2011).
30 NETL. *Carbon Dioxide Capture Handbook*. (U.S. Department of Energy, 2015).
31 Metz, B., Davidson, O., Conick, H. d., Loos, M. & Meyer, L. *IPCC Special Report on Carbon Dioxide Capture and Storage*. (2005).
32 Feron, P. *et al.* Amine Based Post-combustion Capture Technology Advancement for Application in Chinese Coal Fired Power Stations. *Energy Procedia* **63**, 1399-1406, doi:https://doi.org/10.1016/j.egypro.2014.11.149 (2014).
33 Fan, G.-j., Wee, A. G. H., Idem, R. & Tontiwachwuthikul, P. NMR Studies of Amine Species in MEA−CO2−H2O System: Modification of the Model of Vapor−Liquid Equilibrium (VLE). *Industrial & Engineering Chemistry Research* **48**, 2717-2720, doi:10.1021/ie8015895 (2009).
34 Simoes, M. C., Hughes, K. J., Ingham, D. B., Ma, L. & Pourkashanian, M. Predicting Speciation of Ammonia, Monoethanolamine, and Diethanolamine Using Only Ionic Radius and Ionic Charge. *Industrial & Engineering Chemistry Research* **57**, 2346-2352, doi:10.1021/acs.iecr.7b04250 (2018).
35 Team, T. I. E. C. M. Amine-based Post-Combustion CO2 Capture. (Department of Engineering and Public Policy, Carnegie Mellon University, Pittsburgh, PA 15213, 2019).
36 Gjernes, E. *et al.* Results from 30 wt% MEA Performance Testing at the CO2 Technology Centre Mongstad. *Energy Procedia* **114**, 1146-1157, doi:https://doi.org/10.1016/j.egypro.2017.03.1276 (2017).
37 Riboldi, L. & Bolland, O. Overview on Pressure Swing Adsorption (PSA) as CO2 Capture Technology: State-of-the-Art, Limits and Potentials. *Energy Procedia* **114**, 2390-2400, doi:https://doi.org/10.1016/j.egypro.2017.03.1385 (2017).

38 Siqueira, R. M. *et al.* Carbon Dioxide Capture by Pressure Swing Adsorption. *Energy Procedia* **114**, 2182-2192, doi:https://doi.org/10.1016/j.egypro.2017.03.1355 (2017).

39 Shah, G., Ahmad, E., Pant, K. K. & Vijay, V. K. Comprehending the contemporary state of art in biogas enrichment and CO2 capture technologies via swing adsorption. *International Journal of Hydrogen Energy* **46**, 6588-6612, doi:https://doi.org/10.1016/j.ijhydene.2020.11.116 (2021).

40 mundi, i. *Henry Hub Natural Gas Futures End of Day Settlement Price*, <https://www.indexmundi.com/commodities/?commodity=natural-gas&months=60> (2022).

41 Yue, P. *et al.* Life cycle and economic analysis of chemicals production via electrolytic (bi)carbonate and gaseous CO2 conversion. *Applied Energy* **304**, 117768, doi:https://doi.org/10.1016/j.apenergy.2021.117768 (2021).
